# *Cucurbita maxima* Plomo Peel as a Valuable Ingredient for Bread-Making

**DOI:** 10.3390/foods14040597

**Published:** 2025-02-11

**Authors:** Durim Alija, Remigiusz Olędzki, Daniela Nikolovska Nedelkoska, Ewa Pejcz, Agata Wojciechowicz-Budzisz, Viktorija Stamatovska, Joanna Harasym

**Affiliations:** 1 Faculty of Technology and Technical Sciences Veles, University St. Kliment Ohridski-Bitola, Dimitar Vlahov 57, 1400 Veles, North Macedonia; durim.alija@uklo.edu.mk (D.A.); daniela.nedelkoska@uklo.edu.mk (D.N.N.); viktorija.stamatovska@uklo.edu.mk (V.S.); 2 Faculty of Food Technology and Nutrition, University of Tetova, Str. Ilinden, nn., 1200 Tetova, North Macedonia; 3 Adaptive Food Systems Accelerator-Science Centre, Wroclaw University of Economics and Business, 53-345 Wroclaw, Poland; remigiusz.oledzki@ue.wroc.pl; 4 Department of Biotechnology and Food Analysis, Wroclaw University of Economics and Business, Komandorska 118/120, 53-345 Wroclaw, Poland; ewa.pejcz@ue.wroc.pl (E.P.); agata.wojciechowicz-budzisz@ue.wroc.pl (A.W.-B.)

**Keywords:** pumpkin peel, bread-making, antioxidant activity, rheology, functional food, by-product utilization, texture profile, storage stability

## Abstract

The utilization of food industry by-products represents a significant opportunity for developing functional foods. This study investigated the incorporation of *Cucurbita maxima* Plomo peel powder (PS) into wheat bread formulations to assess its potential as a valuable ingredient for bread-making. PS was incorporated into wheat flour at 1%, 10%, and 20% levels. The dough’s rheological properties were analyzed using Mixolab. Bread samples were evaluated for physical characteristics (volume, texture, colour), antioxidant properties (DPPH, ABTS, FRAP), and reducing sugar content. Analyses were performed on day 0 and after 7 days of storage. PS incorporation significantly modified dough rheology, with increased development time and enhanced protein stability. Bread volume decreased progressively with PS addition (from 195.5 cm^3^ to 109.8 cm^3^ at 20% PS). However, antioxidant activity increased substantially, particularly in the crust, with ABTS values rising from 2.37 to 10.08 TE μM/g DM in water extracts. Total phenolic content and reducing sugars showed significant increases across all PS concentrations. Storage studies revealed stable antioxidant properties but progressive textural changes, with hardness increasing from 6.83 N to 108.8 N at 20% PS after 7 days. While PS incorporation affects bread’s physical properties, the significant enhancement in antioxidant activity and phenolic content suggests its potential as a functional ingredient. The optimal incorporation level should balance technological properties with nutritional benefits.

## 1. Introduction

The food industry produces large volumes of waste or by-products, often underutilized despite their nutritional and phytochemical densities. These food fractions have economic potential and could be converted into higher-value products that contribute back to the food supply chain in food waste valorization [1]. Therefore, the agricultural and food byproducts, as inexpensive resources, present new market opportunities for the food, pharmaceutical, and cosmetic industries, and their transformation into sustainable products prevents the loss of valuable nutrients and bioactive components [2].

Pumpkin, a member of the plant genus *Cucurbita*, is an extensively cultivated and widely consumed vegetable, famous for its nutritional value, health-promoting effects and economic benefits. Pumpkin is a good source of nutrients and phytochemicals, such as β-carotene, dietary fibre, vitamins, and minerals [3,4,5,6]. Also, pumpkin has recently gained much attention due to its health-promoting effects, including antidiabetic, antioxidant, anticancer, anti-inflammatory, antihypertensive and cardioprotective, and anti-hyperlipidemic and antimicrobial effects [7]. Because of its nutritional value, large quantities of pumpkin are processed in the food industry into various food products for the needs of the bakery, meat, dairy and beverage industry [8,9,10]. Industrial processing of pumpkin produces large quantities of waste biomass, mainly in the form of peel and seeds. These by-products are rich sources of nutrients and bioactive components and can be utilized as food or incorporated into other foods as functional ingredients [11]. Processing generally produces about 72–76% pumpkin flesh, 2.6–16% peels and 3.1–4.4% seeds [12]. Pumpkin peel is considered a primary pumpkin waste in the food industry because the other two main pumpkin fractions, the flesh and the seeds, are used as ingredients/raw materials in food production or in the form of pumpkin seed oil.

Several studies have investigated the nutritional composition and bioactive profile of pumpkin fractions. The content of nutritional and bioactive compounds depends significantly on the nature of the pumpkin varieties and the influence of environmental conditions [9,13]. However, several studies have reported higher amounts of proteins, ash, fat, and fibre in the pumpkin peel than in the pumpkin flesh [13,14,15]. Pumpkin peels are reported to have the highest amount of beta-carotene and the highest concentration of the amino acid tryptophan compared to the flesh and seeds fractions [16]. Furthermore, pumpkin peel extracts have demonstrated antibacterial and antioxidant activity. In contrast, dietary fibres such as pectin from pumpkin peels have been reported to slow down starch digestion [17] and have a growth-promoting effect on helpful intestinal bacteria [18]. These unique peel characteristics indicate that this part of the pumpkin fruit could be utilized to develop sustainable and innovative functional food products. Staichok et al. [10] reported that partial substituting wheat flour with pumpkin (*Cucurbita moschata*) peel flour in bread influenced their mass growth, colour, and raw fibre and protein content.

Recognizing that food industry by-product utilization presents opportunities for sustainable ingredient development, this research characterized the impact of pumpkin peel (*Cucurbita maxima* Plomo) powder incorporation (1%, 10%, and 20%) into soft wheat flour on dough rheology, bread physicochemical properties, and functional attributes. Bread samples were evaluated for rheological behaviour, physical characteristics, antioxidant properties, and reducing sugar content, while mid-IR spectroscopy analyzed molecular interactions in the bread matrix. Analyses performed at day 0 and after 7-day storage provided comprehensive insights into fibre content impact on dough development, product structure, and antioxidant stability.

## 2. Materials and Methods

### 2.1. Flour Preparation and Ingredients

The base ingredient was soft wheat flour (SWF) (T 750, Stoisław Mill, Stoislaw, Poland) containing 1.8% fat (0.4% saturated fat), 68% carbohydrates, 12% protein, 2.9% fibre, and 0.64% ash. The pumpkin peel (PS) powder derived from *Cucurbita maxima* Plomo (41.95′ N, 20.93′ E, Kamenjane, North Macedonia) contained 2.14% fat, 14.29% protein, 30.77% carbohydrates, and 46.79% total dietary fibre (10.29% soluble and 36.50% insoluble fibre), and 6.01% ash. The pumpkin peel powder was produced by washing the pumpkins and then grating the peel with a household grater. The grated peel was dried in an oven at 45 ± 2 °C for 24 h until reaching less than 10% moisture content. Finally, the dried peel was processed into flour (HC-350, Chemland, Stargard, Poland). The SWF and PS samples were stored in vacuum-sealed containers at ca. 4 °C. The flour blends were prepared using a rotating drum mixer (TM100, Vevor, Guangzhou, China). Three blends were prepared: 1% PS with 99% SWF, 10% PS and 90% SWF, 20% PS and 80% SWF. Pure SWF served as a control sample. Using the AACC 44-19.01 method [19], the moisture content was determined for each sample: the control SWF—10.8%; the blends—9.56% (1% PS), 9.85% (10% PS), and 9.58% (20% PS); and pure PS—9.13%.

### 2.2. Mixolab Dough Analysis

The dough’s rheological properties were analyzed using (Mixolab 2, Chopin Technologies, Paris, France) following the AACCI Method 54-60.01 [20]. The analysis utilized the (Chopin+) protocol over 45 min to assess key parameters, including dough development time, stability, softening, peak viscosity, enzyme activity, and starch retrogradation. Each test utilized 85 g of dough with a fixed 100% water absorption. The process began with an 8 min mixing phase at a constant 30 °C, then heating to 90 °C at 4 °C per minute. The temperature was maintained at 90 °C for 7 min to evaluate heat effects on dough stability, then cooled to 50 °C at the same rate, with a final 5 min holding period. Throughout this process, specific points (C1–C5) were monitored to measure torque, temperature, and time changes, providing data about the dough’s structural behaviour, enzyme activity, and retrogradation potential under thermal conditions.

### 2.3. Breadmaking Process

Breads were elaborated using 2% salt, 2% yeast, and 55% water (per flour weight). Five different samples were prepared: 100% SWF (control), 99:1 (*w*/*w*) SWF/PS flour, 90:10 (*w*/*w*) SWF/PS flour, and 80:20 (*w*/*w*) SWF/PS flour. All the bread used 2% salt, 2% yeast, and 55% water (calculated per flour weight). The recipes were developed based on prior expertise and initial testing. The dough was kneaded for 5 min in a 1000 W dough mixer at speed level 5 (MUM58231, BOSCH, Stuttgart, Germany). Approximately 100 g of dough was placed into bread trays for shaping. After the dough was divided into trays, they were proofed at 35 °C for 1 h at 82% humidity. After 30 min of baking at 180 °C in a steam-convection oven (HENDI 227077, Rhenen, The Netherlands), the loaves were left to cool down for an hour. Samples were evaluated 1 h after baking and after 7 days of storage in a refrigerator (4 °C) in air-tight containers. Each formulation produced four trays, giving four different types of bread—control, 1%, 10% and 20% with pumpkin peel flour (four replicates for each).

### 2.4. Bread Quality Features

#### 2.4.1. Volume and Surface Area

The volume (v) of bread loaves was measured using a 3D scanner (Matter and Form v2, Toronto, ON, Canada) one hour after baking. Three-dimensional scans were taken using the Quick scan tool, and the volume and surface area were calculated by MFStudio +Quickscan (Matter and Form v2, Toronto, ON, Canada) software in triplicate.

#### 2.4.2. Specific Volume and Baking Loss

Specific volume was calculated using volume and weight measurements (Section 2.4.1), according to the formula (Equation (1)):

(1) $$Specific\;volume\;\left[\%\right]=v/w\left[{cm}^{3}/g\right]$$

Baking loss was measured by weighing the samples before baking (W_s_), and 1 h after baking (W_b_), and weight loss (W_w_) was measured after 7 days. The following formula (Equation (2)) was used to obtain the baking/weight loss percentage:

*Baking loss/Weight loss* [%] = [(W_s_ − W_b/w_)/W_s_] × 100 (2)

#### 2.4.3. Physicochemical Characterization of Bread

The bread’s water activity (aw) was measured using an AquaLab 3TE analyzer (Decagon Devices, Inc., Pullman, WA, USA). After carefully separating the bread’s crust and crumbs using a knife, the colour of each was measured using a reflective spectrophotometer (TS7036, #nh, Guangzhou, China). The colour parameters L*, a*, b*, Chroma, hue angle, browning index and whitening index were calculated for the bread’s crust and crumb.

The following formulas were applied to determine the whiteness index (*WI*) (Equation (3)) and the browning index (*BI*) (Equation (4)) for crumb and crust:

(3) $$WI=100-\sqrt{{\left(100-L^{*}\right)}^{2}+a^{{*}^{2}}+b^{{*}^{2}}}$$

(4) $$BI=\frac{100\times (x-0.31)}{0.172}$$

where $x=\frac{a^{*}+1.75L^{*}}{5.645L^{*}+a^{*}-3.012b^{*}}$

#### 2.4.4. Bread Texture Profile Analysis

As previously reported in Oledzki et al. [21], the texture of the bread was assessed using the TPA (texture profile analysis) test using an AXIS texture analyzer FC20STAV500/500 (AXIS, Gdansk, Poland) that was included with the programme AXIS FM v.2_18. Hardness (N) was the force at maximal deformation, whereas cohesiveness, springiness, and chewiness were identified from the peaks. Analysis was carried out in quadruplicate. The mean ± standard deviation was used to report the results.

#### 2.4.5. Measurement of Bread Porosity

A bread porosity analysis was performed using the Analyse particles option implemented in ImageJ 1.54 g (NIH, Bethesda, MD, USA) software on cross-section scans as described elsewhere [22,23].

### 2.5. Antioxidant Activity, Total Phenolic and Reducing Sugar Contents

All the bread’s crust and crumb (control, 1%, 10% and 20%) were tested for antioxidant properties. One gram of the material was combined with ten millilitres of distilled water/ethanol and mixed for one hour at room temperature in a laboratory orbital shaker (WU4, Premed, Marki, Poland) to extract antioxidant compounds. The supernatant was collected after centrifuging the mixture for 10 min at 3500× *g* (MPW-350, MPW, Warszawa, Poland). Following that, the extracted materials were utilized to determine antioxidant levels.

The Folin–Ciocalteu method calculated the total phenolic content [24]. In total, 100 µL of Folin–Ciocalteu reagent and 1.58 mL of distilled water were combined with 20 µL of the extract. Following 5–8 min of room temperature incubation, 300 µL of a saturated Na_2_CO_3_ solution was added. To determine the absorbance at 765 nm, the mixture was incubated for 30 min at 38 °C (MLL147, AJL Electronics, Kraków, Poland) (UV-Vis Ultrospec 2000, Pharmacia Biotech, Piscataway, NJ, USA). A standard curve was created for gallic acid, and the amount of polyphenolic chemicals was expressed in milligrammes of gallic acid equivalent per gram of dry matter (DM).

With a few modifications, the DNS (dinitro salicylic acid) method was used to quantify the reduced sugar concentration [25,26]. In total, 0.25 mL of DNS reagent (1% of 3,5-dinitrosalicylic acid solution in 0.4 M NaOH) was combined with 0.5 mL of the extract. After five minutes of incubation in a boiling water bath, the mixture was cooled to 50–60 °C. 3 mL of distilled water was added, and the absorbance at 530 nm was then measured. For each sample, a duplicate analysis was performed. The results were expressed as glucose equivalent (GE) micrograms per gram of dry matter (DM).

The antioxidant capacity of the samples was measured using the DPPH method [27]. With an absorbance of 0.9 ± 0.1 at 517 nm, 1000 µL of 0.1 mM DPPH solution (2,2-diphenyl-1-picrylhydrazyl) in methanol was mixed with 34.5 µL of the extract. The absorbance was measured at 517 nm, following a 20-min incubation period at room temperature. The results were expressed as Trolox (TE) micrograms per dry matter (DM) gram.The anti-radical capacity of bread’s crust and crumb against the cationic radical 2,2-azo-bis (3-ethylbenzothiazoline-6-sulfonic acid (ABTS•+) has been assessed using the Sridhar et al. [28] method. After diluting the ABTS•+ stock solution with phosphate buffer, the sample’s extract was added to the ABTS•+ working solution. A duplicate analysis was carried out for every sample. The results were expressed as Trolox (TE) micrograms per dry matter (DM) gram. With some minor modifications, the FRAP method by Re et al. [29] was used to assess the bread’s crust and crumb samples’ reducing power (ability to decrease ferric ions Fe^3+^). One millilitre of FRAP working solution was mixed with the bread’s extract. A spectrophotometer (SEMCO, S91 E, Warsaw, Poland) was used to measure the absorbance at 593 nm after the mixture had been stirred and allowed to stand at 36 °C for 20 min. A duplicate analysis was carried out for every sample. The results were expressed as iron (II) sulphate equivalent FeSO_4_−·7H_2_O micrograms per dry matter (DM) gram.

### 2.6. Mid-Infrared (MIR) Spectroscopy

Medium-infrared spectra of blends were obtained using an ATR assembly (Nicolet 6700, Thermo Fisher Scientific, Waltham, MA, USA) at the 4000–400 cm^−1^ range and resolution of 2 cm^−1^. Each sample spectra were compared and analyzed using commercial computer software (OriginPro 2021b SR2).

### 2.7. Statistical Analysis

The results’ variance (ANOVA) analysis was evaluated with Statgraphics Centurion software (Centurion XVII.I version, StatPoint Technologies, Inc., Warrenton, VA, USA). ANOVA was performed with a previous normality of checked data using a *p*-value = 0.05 significance level. Three-way ANOVA for factors like day, proofing time, and sample was performed at a *p*-value = 0.05 significance level.

## 3. Results and Discussion

The rheological analysis of dough enriched with pumpkin peel (PS) powder reveals complex interactions between fibre components and the soft wheat flour matrix (Table 1).

In examining the initial dough development phase, the control dough exhibited a characteristic C1 torque of 1.245 Nm at 2.65 min. The incorporation of PS notably influenced dough development time, with 1% PS addition extending it to 6.7 min, while higher incorporation levels paradoxically led to faster development. Interestingly, the mixing stability point remained constant at 8 min across all PS concentrations, suggesting a consistent final hydration state despite varying fibre content. The significant extension of dough development time at lower PS concentrations (1%) reflects slower gluten network formation due to competitive water binding between fibre and gluten, consistent with observations by Aljobair (2024) [30]. However, at higher PS levels, the paradoxical decrease in development time suggests that excessive fibre content may hinder effective hydration and interaction, a phenomenon also reported for pumpkin seed flour by Melese and Keyata (2023) [31].

PS addition strengthened the dough structure during the protein weakening phase, marked by the C2 parameter. The control’s C2 value of 0.649 Nm increased progressively with PS addition, reaching 1.086 Nm at 20% incorporation. This enhancement suggests significant fibre–protein interactions and improved water binding capacity, effectively supporting dough structure during thermal stress. The constant mixing stability across all PS concentrations is noteworthy, as it suggests that fibre addition does not disrupt the final hydration state of the dough. This finding contrasts with studies such as Chikpah et al. (2023) [32], which report decreased stability in fibre-enriched doughs due to disrupted gluten–starch interactions. The enhanced protein weakening phase (C2 parameter) with PS addition corroborates the strengthening of the dough matrix through fibre–protein interactions, as noted by Hoxha et al. (2023) [33]. These interactions likely enhance the dough’s water-binding capacity and resistance to thermal stress.

The starch gelatinization behaviour, represented by C3 values, showed modest increases from the control’s 1.976 Nm to 2.083–2.319 Nm with PS addition. This indicates that PS fibres integrate into the matrix without substantially disrupting starch gelatinization processes, potentially even reinforcing the gel structure through complementary interactions. The modest increase in starch gelatinization values (C3) upon PS addition aligns with findings by Badia-Olmos et al. (2023) [34], suggesting that PS fibres integrate into the dough matrix without significantly disrupting starch functionality.

Hot gel stability, measured by C4 values, improved with PS incorporation, increasing from 2.114 Nm in control to 2.409 Nm at 20% PS. This enhancement suggests that PS fibres contribute to structural stability at elevated temperatures, likely through enhanced water management and gel network reinforcement. The consistent improvement in hot gel stability (C4) with PS incorporation is indicative of reinforced gel network structures, as reported for pumpkin-based flours by Aljahani (2022) [35]. This behaviour likely stems from the fibrous components’ ability to manage water distribution during heating.

The retrogradation tendency, indicated by C5 values, revealed more complex behaviour. Compared to the control’s 3.319 Nm, PS addition led to varying effects (3.71, 3.46, and 3.667 Nm for 1%, 10%, and 20%, respectively), indicating intricate interactions between PS fibres and starch during the cooling phase. These variations likely reflect competing mechanisms of water binding between fibre components and starch molecules. The complex retrogradation patterns (C5) observed reflect competing mechanisms of water binding by PS fibres and starch. While some studies, such as Aljahani (2022) [35], have reported reduced retrogradation with pumpkin-derived additives, the variability in C5 values across PS concentrations underscores the intricate balance between fibre content and starch reorganization during cooling.

The kinetic parameters, represented by the alpha, beta, and gamma slopes, provide additional insight into the system dynamics. While protein weakening rates remained relatively stable, the starch gelatinization rate showed an apparent decrease with PS addition, suggesting modified starch–water interactions in the presence of fibre. The minimal variation in gamma values indicates consistent gel breakdown behaviour across formulations.

This comprehensive rheological profile demonstrated that PS was an active ingredient in the dough system, substantially modifying dough development, protein stability, and starch behaviour. These modifications suggest potential improvements in bread structure and stability, though optimal incorporation levels would need to balance these rheological effects with final product quality attributes.

**Figure 1:**
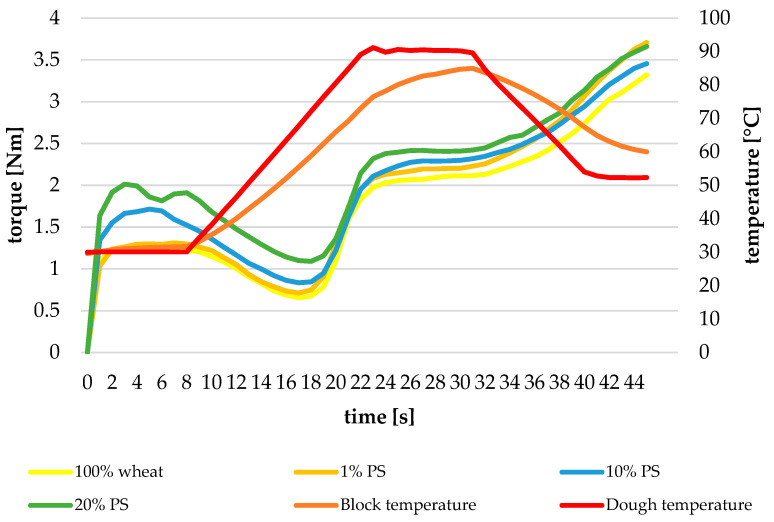
Mixolab profiles of the dough characteristics. PS—pumpkin peel; 1–20%—the share of pumpkin peel powder in blends with soft wheat flour.

The Mixolab profiles in Figure 1 illustrate the thermo-mechanical behaviour of dough enriched with varying pumpkin peel (PS) powder levels.

The Mixolab curves demonstrate distinct patterns across different PS incorporation levels, with each curve characterized by specific torque changes during the mixing, heating, and cooling phases. In the initial mixing phase, all formulations achieve the target torque of approximately 1.1 Nm, indicating successful dough formation. However, the development patterns differ significantly with PS addition.

The control sample (100% wheat flour) exhibits a classical Mixolab profile with well-defined transition points. Upon PS incorporation, notable modifications in dough behaviour become apparent. At 1% PS addition, the curve shows subtle deviations from the control, particularly in the protein weakening phase, suggesting limited interference with gluten network development. This observation aligns with findings by Hoxha et al. (2023) [33], who reported similar effects in fibre-enriched formulations.

As PS concentration increases to 10%, more pronounced changes emerge in the curve profile. The protein weakening phase shows enhanced stability, indicated by a higher minimum torque during heating. This suggests that PS fibres may provide additional structural support during thermal protein denaturation. The starch gelatinization peak becomes slightly broader, indicating modified starch–water interactions in the presence of fibre. Such behaviour is consistent with studies by Badia-Olmos et al. (2023) [34], highlighting the role of fibre in influencing dough thermal transitions.

The most significant alterations are observed with 20% PS incorporation. The curve demonstrates considerably higher torque values during the protein weakening phase, suggesting substantial fibre–protein interactions. The starch gelatinization peak shows reduced amplitude but maintained breadth, indicating that while PS fibres compete for available water, they do not fundamentally disrupt starch gelatinization processes. These findings align with Pereira et al. (2020) [9], which emphasize the stabilizing effects of fibre on dough matrices.

During the cooling phase, all PS-enriched formulations demonstrate modified retrogradation behaviour compared to the control. The final torque values show a concentration-dependent response to PS addition, reflecting complex interactions between fibres, retrograded starch, and the dough matrix during cooling. This behaviour underscores the dual role of PS fibres in reinforcing structure while moderating starch retrogradation, as noted by Aljahani (2022) [35].

These Mixolab profiles reveal that PS incorporation progressively modifies dough behaviour across all mixing–heating–cooling phases, with effects becoming more pronounced at higher incorporation levels. The changes suggest that PS fibres actively participate in dough structure development rather than acting as inert fillers, with implications for final bread quality and stability.

**Figure 2:**
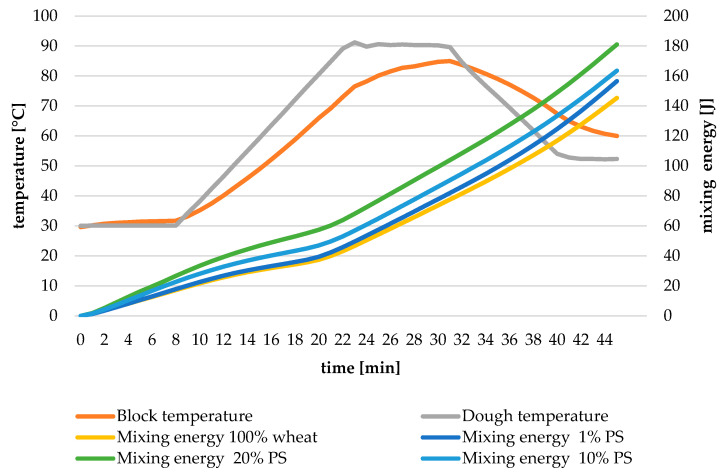
Mixing energy profiles of the dough. PS—pumpkin peel; 1–20%—the share of pumpkin peel powder in blends with soft wheat flour.

Figure 2 presents the mixing energy profiles of dough formulations containing varying levels of pumpkin peel (PS) powder blended with soft wheat flour.

The mixing energy curves demonstrate characteristic variations in energy consumption patterns across different PS incorporation levels. The control sample exhibits a baseline energy profile typical for wheat flour dough. Upon PS addition, notable changes in energy requirements become evident throughout the mixing process. At 1% PS incorporation, the energy profile shows minor deviations from the control, suggesting a limited impact on dough development energetics. The curve maintains similar patterns but with slightly modified amplitude, indicating subtle changes in the work required for dough formation.

Incorporating 10% PS results in more substantial modifications to the energy profile. The curves indicate increased energy requirements during mixing, suggesting higher mechanical work is necessary for proper dough development. This observation aligns with the expected effects of fibre addition on dough formation mechanics.

At 20% PS level, the energy profile exhibits the most pronounced alterations. The curves demonstrate significantly higher energy consumption throughout the mixing phase, reflecting the substantial work required to incorporate the increased fibre content into the dough matrix. The profile shape also shows distinct modifications, indicating fundamental changes in dough development dynamics.

The mixing energy profiles reveal a clear correlation between PS concentration and energy requirements for dough development. These changes reflect the progressive impact of fibre incorporation on dough formation mechanics and suggest the need for adjusted mixing parameters in practical applications. The observed patterns provide valuable insights for process optimization in PS-enriched bread production.

**Figure 3:**
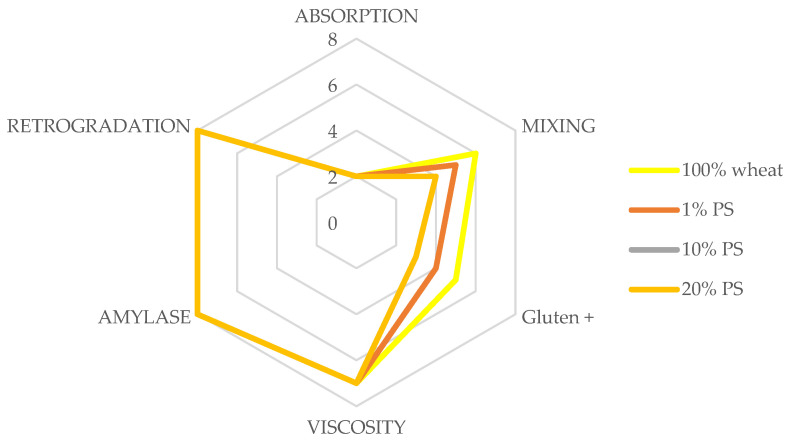
The pumpkin peel powder/soft wheat flour blends characteristics in the dough. PS—pumpkin peel; 1–20%—the share of pumpkin peel powder in blends with soft wheat flour. Absorption-ability of flour to absorb water; mixing-stability of flour during kneading; gluten+-resistance of gluten to heat; viscosity-dough viscosity during heating; amylase-indicates amylase activity; retrogradation-cooked product shelf life.

Figure 3 illustrates the characteristics of pumpkin peel (PS) powder and wheat flour blends in the dough, presented across six key parameters: absorption, mixing, gluten+, viscosity, amylase, and retrogradation.

PS incorporation results in a progressive increase in water absorption, with effects becoming more pronounced as PS content rises from 1% to 20%. This trend reflects the hydrophilic nature of PS fibres, which enhances the composite flour system’s water-binding capacity. These findings align with Aljahani (2022) [35] and Pereira et al. (2020) [9], emphasizing the role of fibre in modifying hydration dynamics.

The mixing parameter analysis reveals variations across PS concentrations, reflecting altered dough stability during kneading. At higher PS levels, the interference of fibre particles with gluten network formation becomes evident. Such behaviour, reported in studies by Hoxha et al. (2023) [33], suggests that the increased fibre disrupts cohesive interactions within the dough matrix.

The Gluten+ values, indicating the gluten network’s resistance to heat, consistently decline with rising PS levels. This pattern suggests that PS fibres interact with the protein matrix, reducing thermal stability. These effects are attributed to fibre–protein interactions and competition for available water, aligning with the findings of Chikpah et al. (2023) [32].

The viscosity parameter demonstrates significant changes during heating, with PS addition modifying the overall rheological behaviour of the dough system. These changes correlate with increased fibre content, influencing the matrix’s water distribution. This behaviour, consistent with Badia-Olmos et al. (2023) [34], underscores fibre incorporation’s structural and functional impact on dough viscosity.

PS incorporation exerts minimal effects on amylase activity, with relatively consistent enzyme activity observed across formulations. This suggests that PS fibres have limited interference with a starch breakdown during mixing and heating.

Conversely, retrogradation behaviour shows notable modifications with increasing PS levels. Altered starch retrogradation patterns indicate that PS fibres influence starch reorganization during cooling and storage. Such effects, documented by Aljahani (2022) [35], have significant implications for PS-enriched bread’ shelf life and textural properties.

The mixing energy profiles and flour blend characteristics provide critical insights into optimizing formulation and processing parameters for PS-enriched bread production. The progressive increase in energy requirements and modified rheological behaviour highlight the need for tailored mixing strategies, particularly at higher PS levels, to maintain dough quality and process efficiency. Balancing fibre enrichment with functional and mechanical considerations will be essential for developing high-quality, nutritionally enhanced bread products.

Table 2 presents the volume, surface area, specific volume, and bake parameters of bread samples containing varying pumpkin peel (PS) powder levels, measured at day 0 and after 7 days of storage.

The bread volume measurements demonstrate a consistent decline with increasing PS incorporation. The control sample exhibits the highest volume (195.5 cm^3^), followed by progressive reductions to 161.6, 136.0, and 109.8 cm^3^ for 1%, 10%, and 20% PS additions, respectively. This trend persists through the 7-day storage period, with minimal changes in relative proportions.

Surface area measurements follow a similar pattern of reduction. The control sample shows the highest surface area (17.81 cm^2^), with progressive decreases observed with increasing PS concentration. The values decrease to 16.54, 14.84, and 12.83 cm^2^ for 1%, 10%, and 20% PS incorporation, respectively. These measurements remain relatively stable during storage.

Specific volume data exhibit a corresponding decline pattern. The control sample demonstrates the highest specific volume (2.40 cm^3^/g), decreasing to 1.94, 1.64, and 1.36 cm^3^/g with 1%, 10%, and 20% PS addition, respectively. The storage period shows minimal impact on these values. Two factors ANOVA indicates significant differences among samples (*p* < 0.001) for all measured parameters. However, the storage time effect (day) shows no statistical significance, suggesting stable product characteristics during the evaluated storage period. The interaction between sample composition and storage time (day*sample) also demonstrates no statistical significance. These results indicate that PS incorporation substantially affects bread’s physical characteristics, with higher concentrations leading to a more compact structure. The stability of these parameters during storage suggests that PS addition, while affecting initial bread characteristics, does not significantly impact structural stability during short-term storage.

Aljobair, 2024 [30] also reported a decline in the specific volume of the bread when wheat flour was incorporated into vegetable peel powder (a mix of green pumpkin, watermelon and cucumber peels). Thus, the bread crumb’s cell wall congealing and the composite dough’s reduced ability to retain gas owing to the dilution of the effective concentration of gluten might be responsible for the decrease in loaf volume. This decrease in gluten affected the remaining gluten in the dough, making it less elastic and firm, reducing the dough’s capacity to store gas [36].

Table 3 presents water activity (Aw), bake loss, and porosity parameters of bread with varying pumpkin peel (PS) concentrations, evaluated at day 0 and after 7 days of storage.

Water activity analysis reveals a notable pattern. The control sample shows an Aw of 0.954, with values increasing slightly to 0.960 at 1% PS, then progressively decreasing to 0.946 at 20% PS incorporation. After 7 days, the most significant decline in Aw is observed in 20% PS samples (from 0.946 to 0.926), indicating enhanced moisture redistribution with higher fibre content.

Bake loss measurements demonstrate interesting variations. The control sample shows 16.97% bake loss, with lower values for 1% and 10% PS (15.78% and 15.99%, respectively). However, 20%PS samples exhibit increased bake loss (17.39%), suggesting modified water retention dynamics at higher fibre concentrations. These trends correlate inversely with the volume measurements from Table 2, indicating that samples with higher bake loss generally demonstrate lower volumes.

These observations align with previous studies by Davoudi et al. (2020) [37], which reported that adding pumpkin powder to Taftoon bread improved the overall quality attributes, including moisture retention. The research highlighted that pumpkin powder could positively influence the baking process and alter the final product’s baking loss and water activity. This is corroborated by the work of Kampuse et al. (2015) [38], who reported that the incorporation of pumpkin by-products significantly increased the nutritional quality of wheat bread, which may also affect moisture retention and baking loss.

Porosity measurements show a declining trend with increasing PS concentration. The control sample exhibited 55.67% porosity, slightly increasing to 58.99% in the 1% sample (statistically insignificant) and then decreasing in 10%PS (50.88%) and 20%PS (46.18%), respectively. This trend directly aligns with the volume and specific volume data from Table 2, where higher PS concentrations result in a more compact structure with reduced porosity. The absence of detailed information about the granulometric distribution of pumpkin peel powder hinders a more comprehensive comparison. However, Niño-Medina et al. (2019) [39] in his study investigated the effects of dietary fibre from chickpea and soybean husk byproducts as baking additives, where they observed that the addition of soluble dietary fibre could significantly influence the texture and volume characteristics of the final product. This suggests that incorporating pumpkin peel powder, which is rich in dietary fibre, could similarly enhance the porosity of composite bread by affecting the dough structure and gas retention during baking [39].

These similarities suggest an interconnected relationship between structural and moisture-related parameters. The reduced volume and specific volume observed in Table 2 correspond to decreased porosity and modified water activity patterns in Table 3. This relationship becomes more pronounced with increasing PS concentration, indicating that fibre incorporation simultaneously affects the bread matrix’s structural development and moisture distribution.

Two-factor ANOVA reveals significant effects (*p* < 0.001) for sample composition on all parameters, with notable sample*days interactions for water activity and porosity. These interactions suggest that PS concentration influences initial bread characteristics and their evolution during storage, particularly regarding moisture distribution and structural stability.

Table 4 presents the texture profile analysis of composite bread with varying pumpkin peel (PS) concentrations, evaluated at day 0 and after 7 days of storage.

The hardness parameter shows significant variation in PS concentration and storage time. The control sample exhibits an initial hardness of 6.83 N, which increases to 32.3 N after 7 days. At 1% PS, similar values are observed (10.23 N increasing to 38.3 N). However, 10% PS shows notably higher initial hardness (14.35 N, increasing to 50.9 N). The most significant change is observed at 20% PS, where an initial hardness of 31.71 N increases substantially to 108.8 N after storage, indicating complex fibre–matrix interactions affecting texture development. The increase in breadcrumb hardness under the influence of the addition of PS agrees with the results found by Davoudi et al. (2020) [38], who reported that after the pumpkin powder was added, the shear stress and, thus, the bread staling increased. Różyło et al. (2014) [40] confirmed that including pumpkin pulp increased bread crumb hardness and chewiness, emphasizing the textural impact of fibre-rich ingredients in bread formulations. According to Minarovičová et al. (2018) [41], incorporating pumpkin powder increased the wheat rolls’ firmness two hours after baking. Furthermore, the firmness of the rolls continued to rise during storage. The hardening effect observed after adding dietary fibre is attributed to the dilution of gluten content and the thickening of the walls surrounding the air bubbles in the crumb. However, opposite results were obtained by Nyam et al. (2013) [42], where the hardness of bread was reduced by adding pumpkin rind flour to bread formulas. According to Staichok et al. (2016) [10], adding pumpkin peel flour did not affect the hardness or masticability of wheat breads.

Cohesiveness values demonstrate a consistent decline pattern with storage across all formulations. Initial values range from 0.828 in control to 0.693 in 20% PS samples, decreasing to 0.564 and 0.471, respectively, after 7 days. This trend suggests the progressive weakening of the internal structure during storage, with PS concentration influencing the rate of change. Similarly, according to Staichok et al. (2016) [10], the pumpkin peel flour addition caused a decrease in the cohesivity and elasticity of bread.

Springiness measurements show a gradual decrease with increasing PS concentration. The control sample exhibits 0.823 initial springiness, while 20% PS shows 0.684. These values show minimal changes during storage, indicating that PS primarily affects initial structure formation rather than storage stability. Nyam et al. (2013) [42] reported that adding pumpkin rind flour did not affect the springiness or cohesiveness of the bread.

Chewiness values present interesting dynamics. Initial values increase with PS concentration from 4.66 N in the control to 14.86 N in 20% of PS samples. After storage, all formulations show increased chewiness, with 20% PS demonstrating the most substantial increase to 35.03 N, reflecting complex changes in the product’s textural characteristics. However, Nyam et al. (2013) [42] observed that the chewiness of bread samples decreased with the addition of pumpkin rind flour.

Resilience measurements show a general decline during storage across all formulations, with initial values ranging from 0.471 to 0.464, decreasing to approximately 0.33 after 7 days, regardless of PS concentration. In the study by Nyam et al. (2013) [42], the control bread had the highest resilience score of 0.41. The addition of pumpkin rinds to the bread resulted in a decrease in resilience values.

Statistical analysis reveals significant effects (*p* < 0.001) of both PS concentration and storage time on most textural parameters. The sample × days interaction shows significance for hardness and chewiness, indicating that PS concentration influences initial texture and its evolution during storage. These textural changes correlate with the structural modifications observed in previous tables, particularly the reduced volume and porosity, suggesting that PS incorporation fundamentally affects both the macro- and micro-structural characteristics of the bread. Gluten and its ratio to starch are critical factors influencing the stiffness and flexibility of bread. The reduced gluten content caused by replacing wheat flour with PS likely increased the fibre-to-gluten ratio, contributing to greater bread staling. Furthermore, components of PS may promote interactions between amylose and amylopectin chains, leading to accelerated starch retrogradation [37]. Another contributing factor to the increased staling in bread containing PS is reduced moisture content, aligning with findings by Franco et al. (2024) [43], who observed that lower moisture levels generally lead to faster bread staling. Breads with low elasticity are more prone to breaking or experiencing irreversible rupture. Adding PS decreased elasticity, leading to lower elasticity values in both formulations than the standard bread. According to Szczesniak 2022 [44], low cohesiveness signifies that less force is needed to stretch a food until it ruptures. As a result, the bread with 20% PS required less force to rupture than the control, making it more prone to crumbling, especially after 7 days of storage.

**Figure 4:**
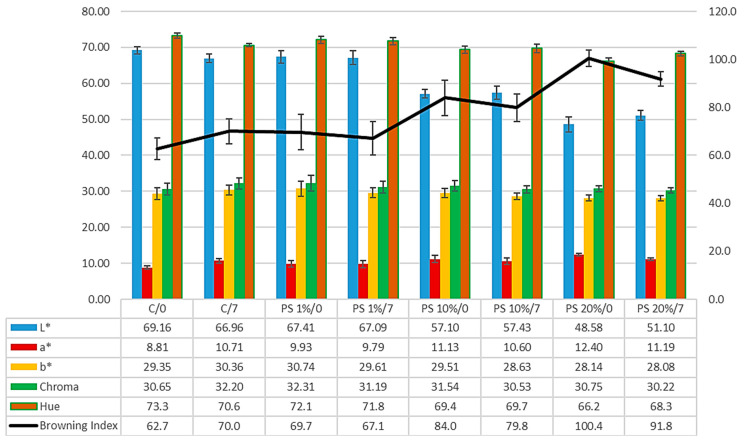
The colour of the bread crust. C—control bread (100% soft wheat flour), 1–20%—the share of pumpkin peel powder (PS) in blends with soft wheat flour, 0 and 7—time points in days after baking.

Figure 4 presents bread crust colour parameters (L*, a*, b*, Chroma, hue angle, and browning index) with varying pumpkin peel (PS) concentrations, evaluated at day 0 and after 7 days of storage.

The L* value (lightness) consistently declines with increasing PS concentration. The control sample exhibits an initial L* of 69.16, decreasing progressively with PS addition to 67.41, 57.10, and 48.58 for 1%, 10%, and 20% PS, respectively. This trend indicates significant darkening of the crust with increased PS content. After 7 days of storage, all samples show slight decreases in L* values, with the most notable change in the control sample (from 69.16 to 66.96). Similar findings were reported by Ghendov-Mosanu et al. (2023) [45], who examined the impact of pumpkin powder addition on the quality and textural properties of shortbread cookies. The lower crust L* values result from browning and Maillard reactions, which are influenced by the content of reducing sugars and proteins on the sample’s surface [46].

The a* value (red-green axis) demonstrates an increasing trend with PS incorporation. The control shows an initial a* of 8.81, increasing to 12.40 in 20% of PS samples, indicating enhanced redness with higher PS concentrations. The values show minor fluctuations during storage, suggesting relatively stable red tones.

The b* value (yellow-blue axis) shows more complex behaviour. Initial values range from 29.35 in the control to 28.14 in 20% PS samples, with variations during storage. These changes suggest complex interactions affecting the yellow component of the crust colour.

Chroma values, representing colour saturation, show slight variations across formulations (30.65–30.75) with minimal changes during storage. This suggests that while PS affects individual colour parameters, the overall colour intensity remains relatively stable.

Hue angle values decrease with increasing PS concentration, from 73.3° in control to 66.2° in 20% of PS samples, indicating a shift in the fundamental colour character of the crust.

The Browning Index shows the most pronounced changes, increasing significantly with PS concentration from 62.7 in control to 100.4 in 20% PS samples at day 0. This trend persists through storage, though with slightly modified values.

Statistical analysis reveals significant effects (*p* < 0.001) for sample composition and storage time on most colour parameters, with notable sample*days interactions. These results indicate that PS incorporation substantially influences initial crust colour development and its evolution during storage.

**Figure 5:**
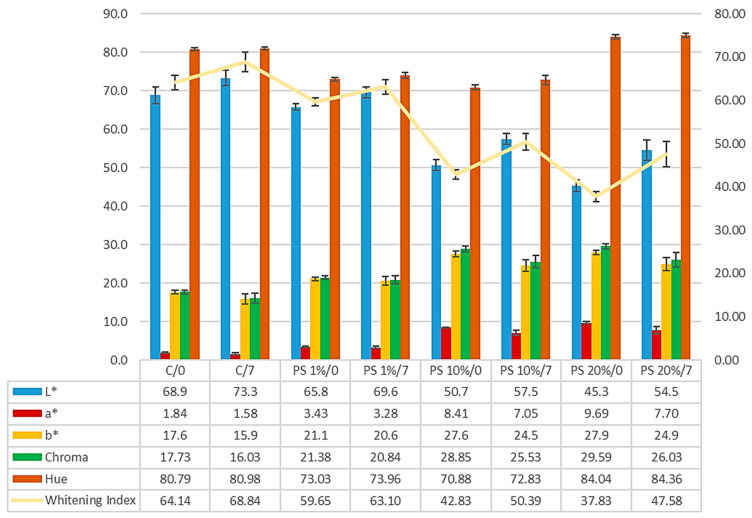
The colour of the bread crumb. C—control bread (100% soft wheat flour), 1–20%—the share of pumpkin peel powder (PS) in blends with soft wheat flour, 0 and 7—time points in days after baking.

Figure 5 presents bread crumbs’ colour parameters (L*, a*, b*, Chroma, hue angle, and whitening index) with varying pumpkin peel (PS) concentrations evaluated at day 0 and after 7 days of storage.

The L* value (lightness) substantially declines with increasing PS concentration. The control sample exhibits an initial L* 68.9, decreasing significantly to 65.8, 50.7, and 45.3 for 1%, 10%, and 20% PS, respectively. Interestingly, all samples show increased L* values after 7 days of storage, with the control reaching 73.3 and 20% PS to 54.5, indicating a lightning effect during storage. Similarly, in the study by Davoudi et al. (2020) [37], the addition of pumpkin powder to Taftoon bread resulted in a darker surface, with its lightness (L*) decreasing from 80.39 in the control sample to 62.04 in the bread containing 5% pumpkin powder. In the work of Nyam et al. (2013) [42], the crumb’s darkness increased proportionally with the rise in pumpkin rind content in the formulation. Similar observations were reported by Rakcejeva et al. (2011) [47], who noted that incorporating pumpkin powder into bread formulations led to a darker appearance. The a* value (red-green axis) shows a progressive increase with PS incorporation. The control displays an initial a* of 1.84, increasing substantially to 9.69 in 20% of PS samples, indicating enhanced redness with higher PS concentrations. After storage, most samples show decreased a* values, suggesting some colour fading. In the studies conducted by Davoudi et al. (2020) [37], the addition of pumpkin powder up to 5% did not significantly affect the bread’s redness (a*). However, increasing the pumpkin powder content to 10% or 15% caused the bread’s colour to shift noticeably towards a reddish hue. In the study by Nyam et al. (2013) [42], the a* values showed that bread with 5% pumpkin rinds (3.15) and 10% pumpkin rinds (3.12) was noticeably more red than the control bread (0.03). The b* value (yellow–blue axis) demonstrates significant variation with PS addition. Values increase from 17.6 in the control to 27.9 in 20% PS samples at day 0, indicating enhanced yellowness with PS incorporation. Storage leads to slight decreases in b* values across all formulations. Similarly, Davoudi et al. (2020) [37] observed that the b* value of bread containing 10% and 15% pumpkin powder was significantly higher than the control bread. Nyam et al. (2013) [42] also observed an increase in bread yellowness. The yellow colour of pumpkin is primarily attributed to its high carotenoid content, as pumpkin is a rich source of β-carotene. The peels contained more β-carotene than the other pumpkin parts [2].

Chroma values increase with PS concentration, from 17.73 in control to 29.59 in 20% of PS samples, indicating enhanced colour intensity. These values show minor reductions during storage, suggesting slight colour fading. The hue angle presents complex behaviour, with initial values ranging from 80.79° in control to 84.04° in 20% PS samples. These changes indicate subtle modifications in the fundamental colour character of the crumb structure. The whitening index consistently declines with increasing PS concentration, from 64.14 in control to 37.83 in 20% PS samples at day 0. All samples showed increased Whitening Index values during storage, with the control reaching 68.84 and 20% PS increasing to 47.58.

The colour changes in the crumb structure show distinct patterns from those observed in the crust, suggesting different mechanisms of colour development and stability between the two bread regions. External appearance and cross-sections of composite breads are shown in Supplementary Figure S1.

Reducing sugar content and antioxidant activity of the crumb and crust of composite breads are shown in Table 5.

The analysis of reducing sugars and antioxidant activity reveals distinct patterns between the crust and crumb parts of the composite bread. The reduced sugar content consistently increases with higher PS incorporation in both bread parts. In the crust, values rise from 1.18 mg/1 g DM in the control to 6.82 mg/1 g DM at 25% PS, while crumb values increase from 1.89 mg/1 g DM to 6.93 mg/1 g DM. This trend suggests that PS addition significantly enhances the availability of reducing sugars, potentially contributing to both antioxidant activity and Maillard reaction development.

Total phenolic content (TPC) measurements using ethanol and water extraction methods demonstrate notable differences between crust and crumb parts. In the crust, ethanol extraction yields increase from 0.02 GAE mg/1 g DM in the control to 1.07 GAE mg/1 g DM at 25% PS, while water extraction shows even more pronounced increases from 0.25 to 5.26 GAE mg/1 g DM. The crumb part exhibits similar trends but generally lower values, suggesting that thermal processing during baking may influence phenolic compound distribution and stability.

The results of the study conducted on yellow pumpkin grown in Indonesia (*Cucurbita moschata*) also showed that the addition of pumpkin flour could significantly increase the total phenolic content (in the enriched bread), which was also significantly dependent on the amount of the added functional additive (pumpkin flour). In the present study, the highest total phenolic content was observed in the bread enriched with 20% pumpkin flour (5.39 mg GAE/g DM), which was 3.9 times higher than the total phenolic content found in the control bread (1.38 mg GAE/g DM) [48].

Similar results related to the increase in the total content of phenolic compounds in bread were obtained in studies in which powder resulting from the combination and simultaneous grinding of dried green pumpkin, watermelon, and cucumber peels were used, which was used to produce functional bread [30]. In the cited studies, it was observed that the introduction of dried powder from green pumpkin, watermelon and cucumber peels in the amount of 5% and 10% of the bread mass resulted in a significant increase (by 15.08% and 22.16%, respectively) in the total content of phenolic compounds, compared to the control group [30].

The higher total phenolic content observed in aqueous extracts than in ethanol extracts may be due to the large number of flavanols in pumpkin, which are very soluble in water. Analyses carried out using liquid chromatography confirmed that pumpkin peel contains a large amount of polyphenolic substances, such as epicatechin (up to 6.645 μg/g DM) and catechin (up to 6.146 μg/g DM), which are very soluble in water [6]. Additionally, it has been shown that various parts of the pumpkin are characterized by a high total content of condensed tannins (up to 0.15 mg catechin equivalent mg/g DM), which dissolve well in ethanol and even more in water [6].

It can be assumed that the amounts of added pumpkin peel powder (PS) used in our studies (including 10% and 20%) were high enough to increase the total content of phenolic compounds despite the intensive heating (180 °C) of the dough during the baking process. Studies indicate that thermolabile polyphenol fractions, such as flavonols, can be protected by large amounts (21.95 g/100 g DM) of dietary fibre (dietary fibre) [3], which hinder the thermal degradation of polyphenolic substances [49].

Also, in studies in which the dough used (for baking cookies) was enriched with melon peel powder, a significant increase in the total phenolic compound content in the produced cookies was noted even though the dough was baked at a temperature of 180–220 °C [50].

Similar results were obtained when grape pomace was introduced into wheat bread dough, significantly increasing the total phenolic compound content in the resulting wheat bread [51].

The DPPH radical scavenging activity consistently enhances PS incorporation in both extracts. Ethanol extracts of the crust demonstrate an increase from 0.06 to 0.54 TE μM/1 g DM, while water extracts show more substantial improvements from 0.05 to 2.50 TE μM/1 g DM. The crumb part follows similar patterns but with lower overall values, indicating that the crust part maintains higher antioxidant activity, possibly due to the formation of Maillard reaction products during baking.

Studies conducted on yellow pumpkin (grown in Indonesia) have shown that adding pumpkin flour (in the amount of 5–20%) can significantly increase (even by 20%) the antioxidant activity (measured by DPPH) in the entire volume of bread [48].

Also, in studies conducted by Aljobair 2024 [30], it was shown that the antiradical activity (measured by the DPPH method) in bread was higher compared to the control bread when it was composed of a powder made by mixing ground dried green pumpkin (*Cucurbita maxima* L.) peels, dried watermelon (*Citrullus lanatus* Tunb.) peels, and dried cucumber (*Cucumis sativus*) peels [30].

ABTS radical scavenging capacity demonstrates robust responses to PS addition. In the crust, ethanol extracts increase from 0.14 to 2.29 TE μM/g DM, while water extracts show dramatic improvements from 2.37 to 10.08 TE μM/g DM. The crumb part exhibits lower but significant increases, with water extracts showing powerful enhancement from 0.92 to 7.13 TE μM/g DM.

Studies conducted on yellow pumpkin (grown in Indonesia) have shown that adding pumpkin flour (in the amount of 5–20%) can also significantly increase (even by 136.62%) the antioxidant activity of bread (measured using ABTS) and the intensity of the increase in antioxidant activity was significantly dependent on the amount of added pumpkin flour [48].

It has been confirmed that pumpkin flour, in addition to large amounts of β-carotene soluble in nonpolar solvents, is also a rich source of such bioactive substances as caffeic acid, gallic acid, p-coumaric acid, and ferulic acid, which are soluble in polar solvents, both in water and alcohol. Therefore, these antioxidants may be responsible for increasing the antioxidant activity of the bread we make after adding pumpkin peel flour to its composition [8].

The higher antioxidant activity (measured by both the ABTS and DPPH tests) observed in the bread crust (than in the bread crumb) may be due to the presence of Maillard reaction products, which are formed in the bread crust as a result of the reaction between amino acids (e.g., histidine, Hys) and reducing sugars (e.g., glucose) during the baking of the dough [52]. As a result of this process, melanoidins are formed, which exhibit antioxidant activity and thus increase the anti-free radical activity of the resulting product (including concerning peroxide radicals) [49]. This conclusion is inconsistent with our observed higher value of total reducing sugars in the crust of the resulting composite bread (compared to the crumb of the produced bread).

The ferric reducing power (FeSO_4_) measurements confirm the overall antioxidant enhancement pattern. Crust values in ethanol extracts increase from 0.06 to 0.95 mg/g DM, while water extracts show more substantial improvements from 0.24 to 4.80 mg/g DM. The crumb part demonstrates similar trends but generally lower values, consistent with other antioxidant measurements.

The observed increase in the reducing capacity of the composite bread obtained (due to the addition of pumpkin peel flour may also be related to the presence of a large number of mineral substances in this raw material, such as calcium, potassium, magnesium, iron and selenium [48].

In addition, individual parts of the pumpkin (mainly the peel and pumpkin seeds) also contain trace elements such as copper, zinc and manganese, which are minerals that not only have reducing properties but also, as in the case of zinc, act as cofactors for antioxidant enzymes such as superoxide dismutase (SOD) [53].

Thus, adding pumpkin flour to the production of functional bread may constitute an element that additionally increases the potential antiradical activity of the product (as a result of its consumption) due to the role of zinc (Zn) as a component of endogenous enzymatic antioxidant defence mechanisms (plasma (pSOD) and erythrocyte (eSOD) superoxide dismutase) of the consumer’s body [54].

The statistical significance of these interactions (*p* < 0.001 for most parameters) is visually confirmed by the interaction plots’ non-parallel and often intersecting lines, indicating that PS addition’s effect on antioxidant activity varies substantially between crust and crumb regions. These visual patterns support the numerical findings and provide clear evidence that the antioxidant enhancement effect of PS incorporation is not uniform throughout the bread structure but is notably influenced by the thermal processing conditions experienced by different bread regions during baking.

**Figure 6:**
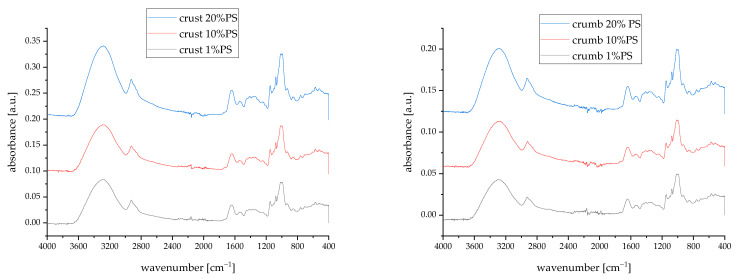
Medium IR spectra of bread crust and crumb. 1–20%—the share of pumpkin peel powder (PS) in blends with soft wheat flour. All samples in the high-frequency region (3600–3200 cm^−1^) show a broad absorption band centred around 3300 cm^−1^, characteristic of O-H stretching vibrations from water molecules, starch, and proteins. The region between 3000 and 2800 cm^−1^ shows characteristic lipid-related CH2 and CH3 stretching bands. These peaks are more pronounced in samples with higher pumpkin peel content, consistent with the lipid contribution from the pumpkin peel we observed in the raw material analysis. Several significant changes are observed in the fingerprint region (1800–900 cm^−1^). The amide I band (around 1650 cm^−1^) and amide II band (around 1550 cm^−1^) are present in all samples, reflecting protein content from wheat flour and pumpkin peel. The intensity of these bands increases with higher pumpkin peel content, particularly noticeable in the 20% PS samples. The complex pattern in the 1000–1200 cm^−1^ region, identified as characteristic of polysaccharides, shows notable differences between crust and crumb samples. The crust samples display more intense and slightly shifted peaks in this region, likely due to starch gelatinization and Maillard reaction products formed during baking. This is particularly evident in the 20% PS crust sample.

The IR spectra of bread crust and crumb samples containing 1%, 10%, and 20% pumpkin peel powder (Figure 6) show characteristic absorption patterns that reflect the chemical changes occurring during the baking process and the influence of pumpkin peel incorporation.

Compared to the raw material spectra (Figure S3) the bread samples show broader, less resolved peaks, indicating the formation of more complex structures during baking. The characteristic pumpkin peel markers we identified previously (carotenoid bands at 960–970 cm^−1^, pectin-related peaks at 1720–1740 cm^−1^) are still detectable but modified, suggesting that while these compounds are incorporated into the bread matrix, they undergo some transformation during baking. The spectral features align well with the compositional data, showing how the incorporation of pumpkin peel powder, with its substantial fibre and protein content, modifies the molecular structure of the bread matrix. The differences between crust and crumb spectra reflect the physicochemical changes occurring during baking.

The FTIR spectroscopic analysis of bread samples with varying levels of pumpkin peel supplementation (PS) reveals characteristic patterns related to acrylamide formation. The analysis focused on the spectral region between 1490 and 1699 cm^−1^, which has been identified by Rexhepi et al., 2024 [55] as optimal for acrylamide quantification due to minimal interference from other food matrix components. The bread samples’ most significant acrylamide-related absorption bands were observed at 1671 cm^−1^, corresponding to the carbonyl (C=O) stretching vibration, and at 1613 cm^−1^, attributed to NH_2_ deformation. Additional characteristic bands include CH_2_ rocking vibrations at 1429 cm^−1^, C-H bending at 1351 cm^−1^, and C-N stretching at 1280 cm^−1^. These assignments are consistent with previous studies on acrylamide detection in thermally processed foods by Ayvaz and Rodriguez-Saona [56]. Considering the amino acid composition data [57], pumpkin peel contains aspartic acid (8.49 mg/g) but notably lacks asparagine, which is required for acrylamide formation through the Maillard reaction. Aspartic acid, while structurally similar, does not participate in acrylamide formation in the same way as asparagine. Therefore, despite the increasing reducing sugar content observed with higher PS addition (from 4.97 to 7.46 mg/1 g DM in blends and corresponding increases in bread crust and crumb), the absence of asparagine in the pumpkin peel suggests that the spectral changes and increased band intensities at 1671 cm^−1^ and 1613 cm^−1^ may not be directly attributable to acrylamide formation. Complementing results were observed by Alija et al. (2025), who verified the acrylamide content in soft wheat bread supplemented with *Cucurbita maxima* Plomo flesh powder [22].

## 4. Conclusions

Incorporating pumpkin peel powder into wheat bread formulations significantly influences the final product’s technological and functional properties. Rheological analysis revealed that PS addition modifies dough development dynamics with notable effects on protein stability and starch behaviour. While higher PS concentrations (20%) led to increased dough development time and modified starch gelatinization patterns, these changes did not compromise dough stability, suggesting the successful integration of PS fibre into the wheat matrix.

Physical characteristics of the bread showed concentration-dependent effects, with increased PS levels leading to reduced volume and modified textural properties. The specific volume decreased from 2.40 cm^3^/g in control to 1.36 cm^3^/g at 20% PS, while crumb hardness significantly increased, particularly during storage. However, these structural modifications were accompanied by enhanced functional properties, particularly evident in antioxidant activity measurements.

PS incorporation significantly improved antioxidant properties, with differential effects observed between crust and crumb regions. The crust demonstrated enhanced antioxidant activity, with ABTS values increasing from 2.37 to 10.08 TE μM/1 g DM in water extracts at 20% PS addition, suggesting synergistic effects between thermal processing and PS bioactive compounds. Total phenolic content and reduced sugar levels increased substantially, indicating enhanced nutritional value.

These findings demonstrate that PS can be successfully incorporated into up to 20% of bread formulations, offering a promising approach to developing functional bakery products while utilizing food industry by-products. While higher PS concentrations affect bread’s physical properties, the significant enhancement in antioxidant activity suggests potential health benefits that may outweigh structural modifications. Future research should optimize PS incorporation levels to balance technological properties with functional benefits, potentially through dough improvers or modified processing conditions.

While comprehensively analyzing the incorporation of pumpkin peel powder (PS) into bread formulations, the research presents several notable limitations. The study focused only on one pumpkin variety (*Cucurbita maxima* Plomo) from a specific geographical location, limiting the generalizability of findings across different pumpkin cultivars and growing conditions. The storage period was relatively short (7 days), preventing long-term stability and shelf life insights. The research lacked sensory evaluation, which is crucial for consumer acceptance and commercial viability. Additionally, the study did not investigate the bioavailability of the enhanced antioxidant compounds or their stability during digestion. The granulometric distribution of the pumpkin peel powder was not detailed, limiting understanding of how particle size affects dough properties. Finally, while acrylamide formation was discussed through FTIR analysis, quantitative measurements of acrylamide levels were not performed, leaving uncertainty about the potential formation of this processing contaminant at different PS concentrations.

## Figures and Tables

**Table 1:** Rheological characteristics of the dough from *Cucurbita maxima* Plomo peel/soft wheat blends.

100% Wheat	Time (min)	Torque (Nm)	Dough Temperature (°C)	C1–C2 C3–C2	C3–C4 C5–C4	Amp. (Nm)	Stability (min)
C1	2.65	1.245	30.9	−14.83 5.52	−7 15.02	0.14	10.3
CS	8	1.224	31.7				0
C2	17.48	0.649	57				
C3	23	1.976	76.5				
C4	30	2.114	84.7				
C5	45.02	3.319	60				
Alpha	−0.102						
Beta	0.566						
Gamma	0.02						
		1% PS					
C1	6.7	1.328	32.7	10.05 6.25	−7 15.02	0.08	9.6
CS	8	1.298	32.8				0
C2	16.75	0.705	57.2				
C3	23	2.083	79				
C4	30	2.203	86.7				
C5	45.02	3.71	59.5				
Alpha	−0.106						
Beta	0.498						
Gamma	0.024						
		10% PS					
C1	4.93	1.721	33.2	−12.19 5.88	−7 15.02	0.11	6.8
CS	8	1.523	33.7				0
C2	17.12	0.828	56.8				
C3	23	2.109	77.5				
C4	30	2.299	85.5				
C5	45.02	3.46	61				
Alpha	−0.09						
Beta	0.38						
Gamma	0						
		20% PS					
C1	3.65	2.032	33	−14.03 5.32	−7 15.02	0.19	8.2
CS	8	1.91	33.9				0
C2	17.68	1.086	60.7				
C3	23	2.319	79.4				
C4	30	2.409	87				
C5	45.02	3.667	59.7				
Alpha	−0.096						
Beta	0.33						
Gamma	0.02						

**Table 2:** Volume, surface area, specific volume, and bake and weight loss on day 0 and after 7 days of storage.

	Volume [cm^3^]		Surface Area [cm^2^]		Specific Volume [cm^3^/g]	
sample	0	7	0	7	0	7
control	195.5 ± 1.0 ^d^	189.9 ± 0.7 ^d^	17.81 ± 0.10 ^d^	18.16 ± 0.10 ^d^	2.40 ± 0.01 ^d^	2.32 ± 0.00 ^d^
1%	161.6 ± 0.8 ^c^	165.6 ± 8.7 ^c^	16.54 ± 0.32 ^c^	16.54 ± 0.32 ^c^	1.94 ± 0.01 ^c^	2.00 ± 0.10 ^c^
10%	136.0 ± 1.0 ^b^	137.5 ± 1.3 ^b^	14.84 ± 0.23 ^b^	14.84 ± 0.23 ^b^	1.64 ± 0.01 ^b^	1.67 ± 0.00 ^b^
20%	109.8 ± 4.0 ^a^	105.1 ± 3.0 ^a^	12.83 ± 0.2 ^a^	12.83 ± 0.02 ^a^	1.36 ± 0.05 ^a^	1.31 ± 0.04 ^a^
day	ns		ns		ns	
sample	***		***		***	
day × sample	ns		ns		ns	

1–20%—pumpkin peel powder addition; lower-case letters mean values in columns are statistically different (*p* = 0.05). ***—statistically different at *p* < 0.001; ns—statistically non-different

**Table 3:** Water activity, bake/weight loss, and porosity of composite breads on day 0 and after 7 days of storage.

	Water Activity (A_w_)		Bake/Weight Loss [%]		Porosity [%]	
sample	0	7	0	7	0	7
control	0.954 ± 0.002 ^b^	0.958 ± 0.006 ^b^	16.97 ± 0.18 ^b^	16.48 ± 0.01 ^a^	55.67 ± 11.57 ^bc^	91.57 ± 3.59 ^d^
1%	0.960 ± 0.002 ^c^	0.957 ± 0.004 ^b^	15.78 ± 0.11 ^a^	16.23 ± 0.16 ^a^	58.99 ± 6.36 ^c^	83.11 ± 2.64 ^c^
10%	0.956 ± 0.001 ^b^	0.954 ± 0.002 ^b^	15.99 ± 0.41 ^a^	16.30 ± 0.71 ^a^	50.88 ± 6.82 ^ab^	71.07 ± 5.88 ^b^
20%	0.946 ± 0.002 ^a^	0.926 ± 0.002 ^a^	17.39 ± 0.76 ^b^	18.60 ± 0.32 ^b^	46.18 ± 4.60 ^a^	67.60 ± 7.50 ^a^
sample	***		***		***	
days	***		ns		***	
sample × days	***		*		***	

1–20%—pumpkin peel powder addition; lower-case letters mean values in columns are statistically different (*p* = 0.05). ***—statistically different at *p* < 0.001; *—statistically different for *p* < 0.05; ns—statistically non-different.

**Table 4:** Texture profile of composite breads on day 0 and after 7 days of storage.

Texture Profile						
Sample	Time (Days)	Hardness [N]	Cohesiveness	Springiness	Chewiness [N]	Resilience
control	0	6.83 ± 1.47 ^a^	0.828 ± 0.060 ^b^	0.823 ± 0.095 ^b^	4.66 ± 1.25 ^a^	0.471 ± 0.025 ^a^
	7	32.3 ± 7.1 ^a^	0.564 ± 0.101 ^a^	0.743 ± 0.024 ^b^	13.39 ± 3.28 ^a^	0.334 ± 0.062 ^a^
1%	0	10.23 ± 0.99 ^ab^	0.783 ± 0.003 ^b^	0.828 ± 0.058 ^b^	6.65 ± 1.06 ^ab^	0.542 ± 0.016 ^b^
	7	38.3 ± 6.1 ^ab^	0.535 ± 0.027 ^a^	0.755 ± 0.036 ^b^	15.37 ± 1.71 ^a^	0.319 ± 0.024 ^a^
10%	0	14.35 ± 2.37 ^b^	0.767 ± 0.022 ^b^	0.746 ± 0.085 ^ab^	8.26 ± 1.93 ^c^	0.493 ± 0.032 ^a^
	7	50.9 ± 8.5 ^b^	0.485 ± 0.060 ^a^	0.711 ± 0.047 ^ab^	17.81 ± 5.60 ^a^	0.322 ± 0.050 ^a^
20%	0	31.71 ± 8.34 ^c^	0.693 ± 0.052 ^a^	0.684 ± 0.051 ^a^	14.86 ± 3.52 ^d^	0.464 ± 0.043 ^a^
	7	108.8 ± 16.8 ^c^	0.471 ± 0.067 ^a^	0.674 ± 0.020 ^a^	35.03. ± 9.97 ^b^	0.333 ± 0.057 ^a^
sample		***	***	**	***	ns
days		***	**	*	***	*
sample × days		***	ns	ns	*	ns

1–20%—pumpkin peel powder addition; lower-case letters mean values in columns are statistically different (*p* = 0.05). ***—statistically different at *p* < 0.001; **—statistically different for *p* < 0.01; *—statistically different for *p* < 0.05; ns—statistically non-different.

**Table 5:** Reducing sugar content and antioxidant activity of composite breads.

		Reducing Sugar	TPC (GAE mg/g DM)		DPPH (TE μM/g DM)		ABTS (TE μM/g DM)		(FeSO_4_ mg/d DM)	
Sample	Part	Glu mg/g DM	EtOH	H_2_O	EtOH	H_2_O	EtOH	H_2_O	EtOH	H_2_O
SWF	crust	1.18 ± 0.00 ^a^	0.02 ± 0.00 ^a^	0.25 ± 0.02 ^a^	0.06 ± 0.00 ^a^	0.05 ± 0.01 a	0.14 ± 0.00 ^a^	2.37 ± 0.09 ^a^	0.06 ± 0.00 ^a^	0.24 ± 0.01 ^a^
1%		1.61 ± 0.06 ^a^	0.43 ± 0.04 ^b^	1.72 ± 0.11 ^b^	0.19 ± 0.06 ^ab^	0.34 ± 0.05 ^b^	1.04 ± 0.08 ^b^	3.69 ± 0.30 ^b^	0.24 ± 0.09 ^b^	0.60 ± 0.03 ^b^
10%		5.26 ± 0.47 ^b^	0.45 ± 0.01 ^c^	2.53 ± 0.17 ^c^	0.29 ± 0.07 ^b^	1.23 ± 0.12 ^c^	1.35 ± 0.09 ^c^	5.92 ± 0.04 ^c^	0.52 ± 0.02 ^c^	2.08 ± 0.20 ^c^
20%		6.82 ± 0.68 ^c^	1.07 ± 0.09 ^d^	5.26 ± 0.14 ^d^	0.54 ± 0.08 ^c^	2.50 ± 0.14 ^d^	2.29 ± 0.14 ^d^	10.08 ± 0.40 ^d^	0.95 ± 0.12 ^d^	4.80 ± 0.24 ^d^
SWF	crumb	1.89 ± 0.02 ^a^	0.03 ± 0.00 ^a^	0.03 ± 0.00 a	0.04 ± 0.00 ^a^	0.04 ± 0.00 a	0.09 ± 0.00 ^a^	0.92 ± 0.07 ^a^	0.01 ± 0.00 ^a^	0.34 ± 0.00 ^a^
1%		3.83 ± 0.47 ^b^	0.46 ± 0.09 ^b^	0.97 ± 0.07 ^b^	0.15 ± 0.02 ^a^	0.49 ± 0.08 ^b^	1.46 ± 0.20 ^b^	2.85 ± 0.18 ^b^	0.12 ± 0.02 ^b^	0.72 ± 0.23 ^b^
10%		5.34 ± 0.40 ^c^	0.44 ± 0.10 ^b^	1.68 ± 0.07 ^c^	0.29 ± 0.03 ^b^	0.82 ± 0.06 ^c^	1.86 ± 0.05 ^c^	5.12 ± 0.31 ^c^	0.24 ± 0.05 ^c^	1.67 ± 0. 19 ^c^
20%		6.93 ± 0.59 ^d^	0.71 ± 0.05 ^c^	2.43 ± 0.05 ^d^	0.42 ± 0.08 ^c^	1.22 ± 0.09 ^d^	1.87 ± 0.20 ^c^	7.13 ± 0.20 ^d^	0.36 ± 0.10 ^d^	2.35 ± 0.04 ^d^
sample		***	***	***	***	***	***	***	***	***
part		**	*	***	ns	***	ns	***	***	***
sample × part		*	**	***	ns	***	**	***	**	***

SWF—soft wheat flour, 1–20%—pumpkin peel powder addition; lower-case letters mean values in columns are statistically different (*p* = 0.05). ***—statistically different at *p* < 0.001, **—statistically different for *p* < 0.01, *—statistically different for *p* < 0.05, ns—statistically non-different.

## Data Availability

The original contributions presented in this study are included in the article/Supplementary Materials. Further inquiries can be directed to the corresponding author.

## Supplementary Materials

The following supporting information can be downloaded at https://www.mdpi.com/article/10.3390/foods14040597/s1, Figure S1: Photos of bread—0 days—NUS. Figure S2. Photos of bread—7 days—NUS. Figure S3: Medium IR spectra of wheat flour (SWF), pumpkin peel powder (PS), and their blends containing 1%, 10%, and 20% PS.
