# Supplementary material for: Cucurbita maxima Plomo Peel as a Valuable Ingredient for Bread-Making
Source: Foods. 2025 Feb 11;14(4):597. doi: 10.3390/foods14040597 (PMC11854699; doi:10.3390/foods14040597)
Supplement: Supplementary file 1 [file foods-14-00597-s001.zip › foods-3430587-supplementary.pdf]

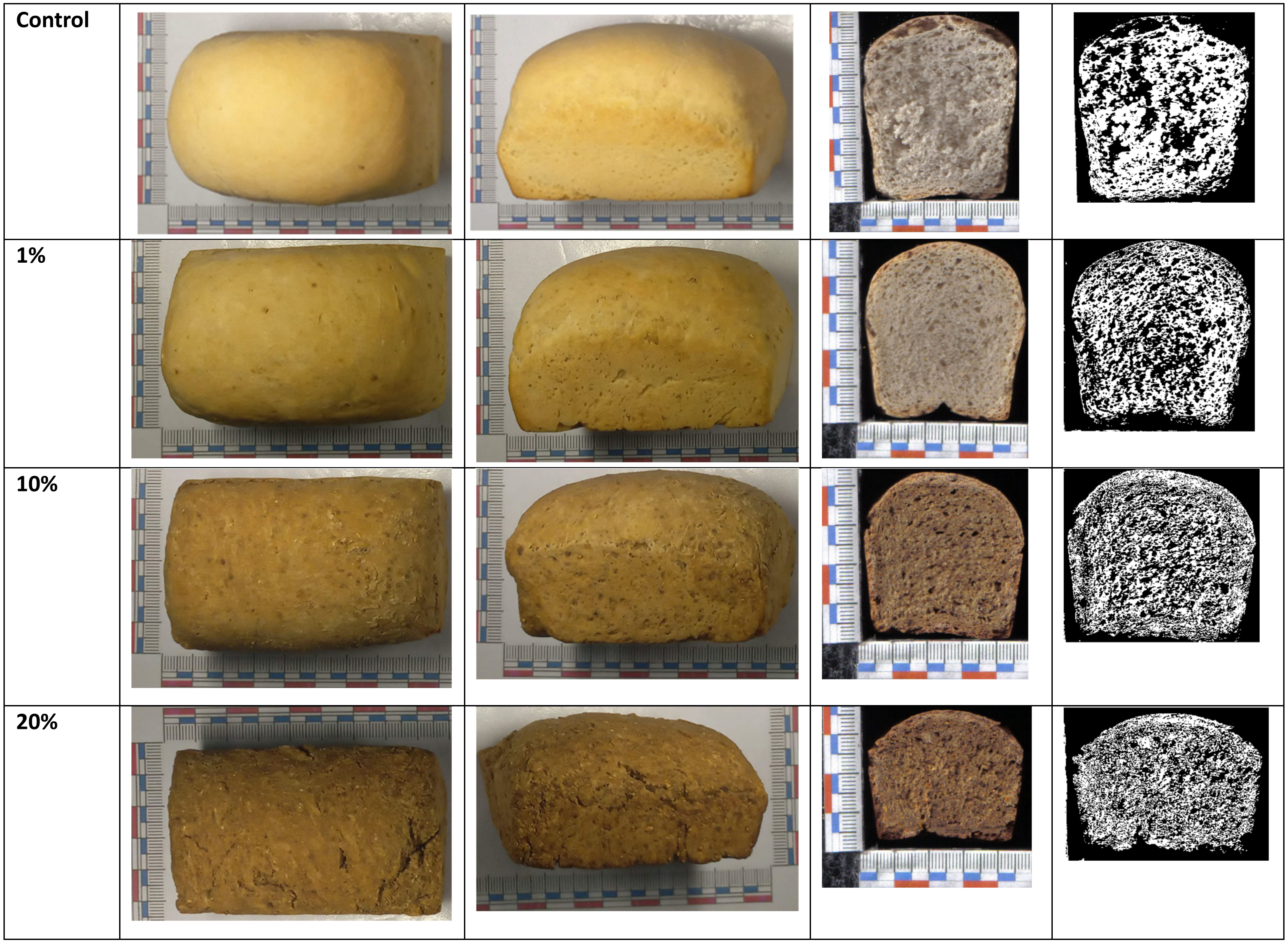

Figure S1. Photos of breads – 0 days – NUS

**Control**	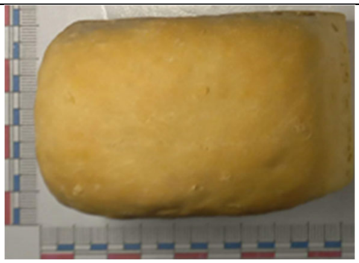	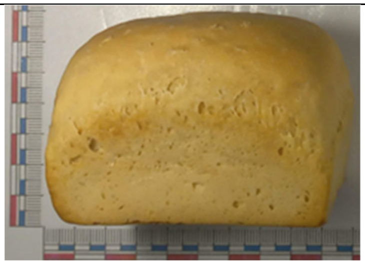	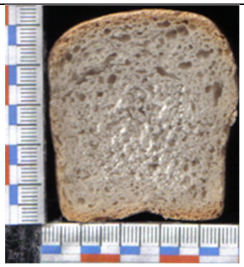	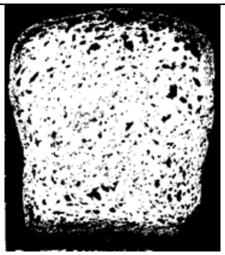
**1%**	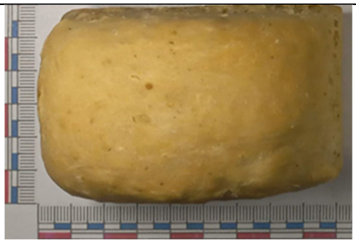	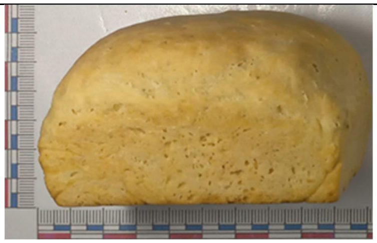	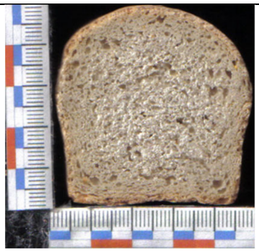	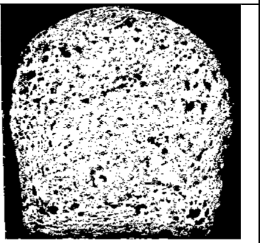
**10%**	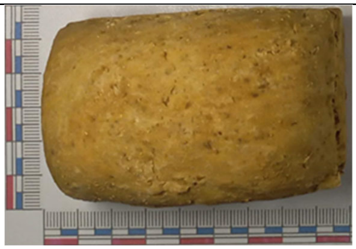	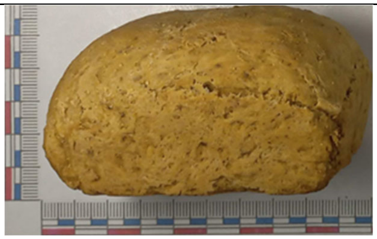	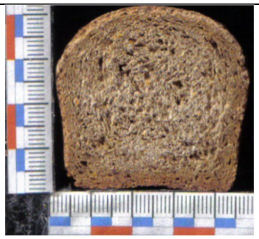	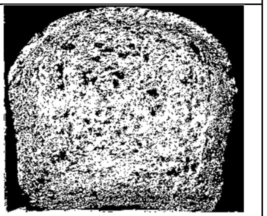
**20%**	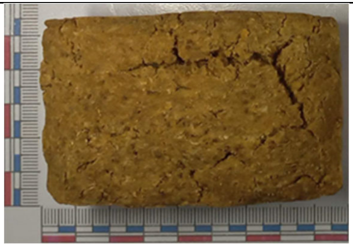	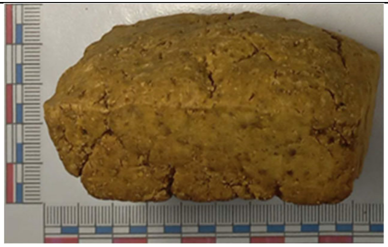	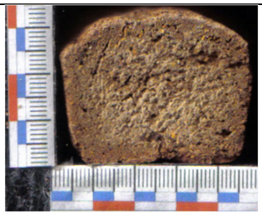	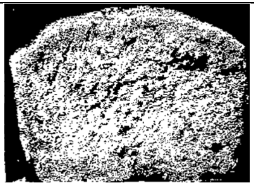

Figure S2. Photos of breads – 7 days – NUS

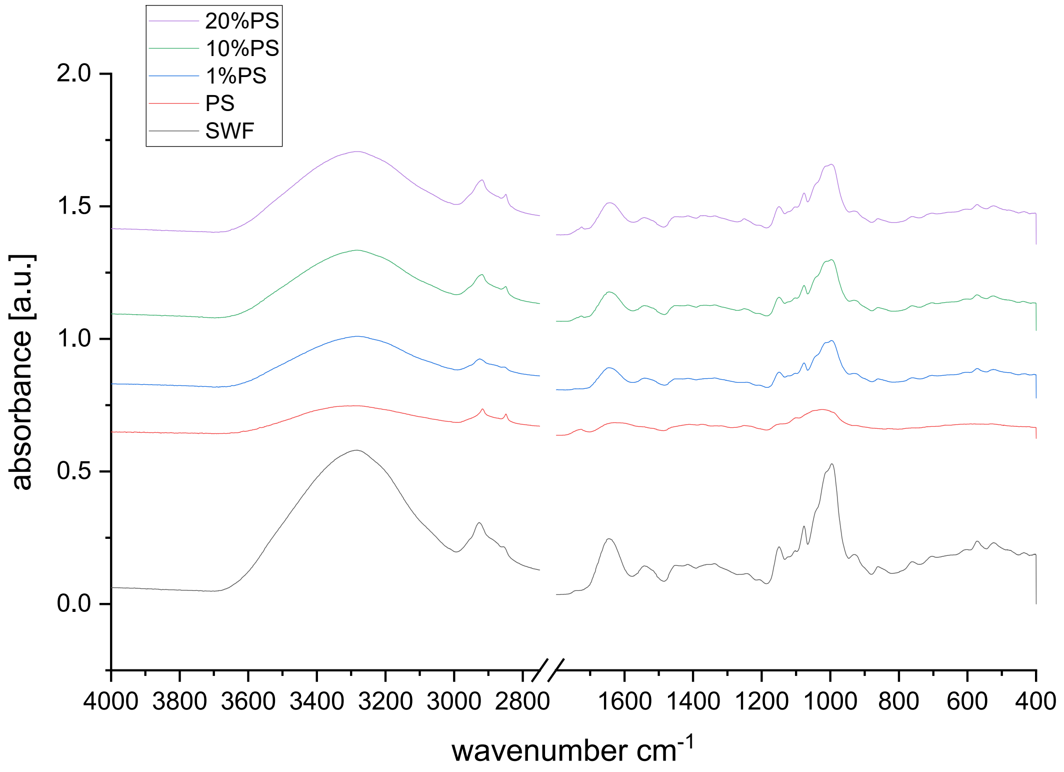

Figure S3. Medium IR spectra of wheat flour (SWF), pumpkin peel powder (PS), and their blends containing 1%, 10%, and 20% PS.
